# Is There a Role for Exercise When Treating Patients with Cancer with Immune Checkpoint Inhibitors? A Scoping Review

**DOI:** 10.3390/cancers14205039

**Published:** 2022-10-14

**Authors:** Jasmine Handford, Miaoqi Chen, Ridesh Rai, Charlotte L. Moss, Deborah Enting, Nicola Peat, Sophia N. Karagiannis, Mieke Van Hemelrijck, Beth Russell

**Affiliations:** 1 Translational Oncology and Urology Research (TOUR), School of Cancer and Pharmaceutical Sciences, King’s College London, London SE1 9RT, UK; 2 Guy’s and St Thomas’ NHS Foundation Trust, London SE1 9RT, UK; 3 St John’s Institute of Dermatology, School of Basic & Medical Biosciences, King’s College London, London SE1 9RT, UK; 4 Breast Cancer Now Research Unit, School of Cancer & Pharmaceutical Sciences, King’s College London, London SE1 9RT, UK

**Keywords:** cancer, immune checkpoint, physical activity, exercise, quality of life, scoping review

## Abstract

**Simple Summary:**

Immune checkpoint inhibitors (ICIs) are drugs which treat cancer by manipulating the immune system. Exercise also influences the immune system and helps to reduce symptoms in people with cancer, particularly fatigue. However, the effect of combining exercise with ICIs has not been well established. We hypothesise that the combined approach will produce beneficial outcomes for people with cancer (such as fewer side effects from ICIs and better killing of cancer cells). To determine the need for, and design of, future studies which address this hypothesis, we first need to understand what previous research has already shown. We aim to identify previous studies which have investigated this topic. Subsequently, by summarising their findings, we aim to communicate the key gaps in current understanding and provide informed recommendations about the direction, and design, of future research addressing the role of exercise during ICI treatment for people with cancer.

**Abstract:**

The impact of using exercise as a non-pharmacological intervention in patients with cancer receiving immune checkpoint inhibitors (ICIs) is not well known. Our objective was to determine the extent of, and identify gaps within, available literature addressing the effect of exercise on (a) oncological outcomes and (b) quality of life (QoL) in patients with cancer receiving ICIs, and (c) the underlying biological mechanisms for such effects. We conducted searches across EMBASE, APA PsycInfo and Ovid MEDLINE(R). Studies were eligible if they addressed at least one aspect of the objective and were available in the English language. Results were synthesised using a narrative approach and subsequently discussed with multidisciplinary stakeholders. As of the final search on 5 April 2022, 11 eligible studies were identified, of which 8 were preclinical and 3 were clinical. Clinical studies only focused on QoL-related outcomes. When studies were grouped by whether they addressed oncological outcomes (n = 7), QoL (n = 5) or biological mechanisms (n = 7), they were found to be heterogeneous in methodology and findings. Additional evidence, particularly in the clinical setting, is required before robust recommendations about whether, and how, to include exercise alongside ICI treatment can be made.

## 1. Introduction

Immunotherapy has become an established treatment modality for patients with cancer in the past decade [1]. Immune checkpoint inhibitors (ICIs), a class of immunotherapeutic agent, comprise monoclonal antibodies which block inhibitory signalling between antigen-presenting cells, such as tumour cells, and T cells. This facilitates T cell activation and subsequent recognition and attack of tumour cells [1]. Research into ICIs has primarily focused on two signalling axes; via CTLA-4 and via PD-1 (and its ligand, PD-L1) [2].

ICIs are approved for use in the treatment of various malignancies including melanoma, Hodgkin’s lymphoma, and non-small cell lung cancer (NSCLC) [2]. Responses to ICIs have been particularly impressive in melanoma, even in the metastatic setting where dual-ICI treatment with the anti-PD-1 (αPD-1) antibody, nivolumab, and the anti-CTLA-4 (αCTLA-4) antibody, ipilimumab, have achieved objective response rates of over 60% [2,3]. However, not all patients respond to ICIs (primary resistance) and a significant proportion of those who do respond, develop resistance to ICI therapies (acquired resistance) [2,4]. Moreover, patients receiving ICIs commonly experience adverse events (74% and 89% of patients receiving PD-1/PD-L1 inhibitors and CTLA-4 inhibitors, respectively), a considerable proportion of which are of high Grade (≥Grade 3), meaning that treatments are often discontinued due to toxicity [5]. Taken together, these challenges highlight the need for interventions which enhance ICI efficacy whilst minimising adverse effects.

According to the World Health Organisation, physical activity (PA) is defined by ‘any bodily movement produced by skeletal muscles that requires energy expenditure’ [6]. Exercise is a subset of PA which is planned, structured, repetitive and has the objective to improve fitness [7]. The American College of Sports Medicine outlines exercise guidelines for patients with cancer [8]. Recommendations state that adults should aim to undertake muscle strengthening sessions twice per week, as well as 150 min of moderate-intensity or 75 min of vigorous-intensity aerobic exercise per week (defined as 40–59% or 60–84% of heart rate reserve, respectively) [6,8,9].

Numerous benefits of exercise for patients with cancer have been documented, including mitigation of cancer- and treatment-related symptoms and improved health-related quality of life (HRQoL) [10,11,12,13]. Studies have also shown a positive effect of exercise on tumour control, with preclinical studies reporting that exercise suppressed tumour growth and rate of recurrence when delivered as a sole intervention [14,15,16,17,18]. Similar trends have been observed in the context of chemotherapy, the largest group of systemic anti-cancer therapies. When combined with chemotherapy, exercise improved quality of life (QoL)-associated factors (including physical functioning and psychological status) and reduced symptom burden (including fatigue, pain, and nausea/vomiting) [19,20]. Exercise also enhanced the response to chemotherapy in the preclinical setting [21], and appeared to associate with improved disease-free survival in breast cancer patients undergoing adjuvant chemotherapy [22]. However, although ICIs comprise an important element of systemic therapy for cancer, the effect of combining ICIs with an exercise intervention is not well understood [13].

Exercise has been shown to modulate the innate and adaptive immune system, including the relative trafficking and activation of different immune cell populations such as NK cells, γδ T-cells, CD4+ T-cells, and CD8+ T cells in the bloodstream and tumour immune microenvironment (TIME) [23,24]. Exercise-dependent changes in cytokine and hormone profiles have also been reported [24]. However, immune responses appear varied depending on the duration (e.g., acute versus chronic), intensity (e.g., low versus moderate versus vigorous) and type (e.g., aerobic versus resistance) of exercise [24,25].

Given the evidence for exercise-dependent effects on tumour control, symptom burden and immune function, we hypothesise that exercise will positively influence the balance between treatment efficacy and adverse events in patients undergoing therapy with ICIs. Determining the need for, and design of, future studies investigating this hypothesis requires a prior understanding of the current research landscape. As such, we conducted a scoping review on this topic, given the purpose of such reviews is to identify the extent of available evidence and potential gaps in the literature [26]. 

Therefore, the scoping research questions were defined to be the following:

1. Is there any evidence that suggests that exercise has a demonstratable effect on improving the oncological outcomes of patients with cancer receiving ICIs?
2. Is there any evidence that suggests that exercise (including which type, timing and dosage of exercise) has a demonstratable effect on improving the QoL of patients with cancer receiving ICIs?
3. What are the biological mechanisms, if any, that could be responsible for the effects exerted by exercise on improving the oncological outcomes and QoL of patients with cancer receiving ICIs?

## 2. Materials and Methods

### 2.1. Protocol and Reporting

**Figure 1:**
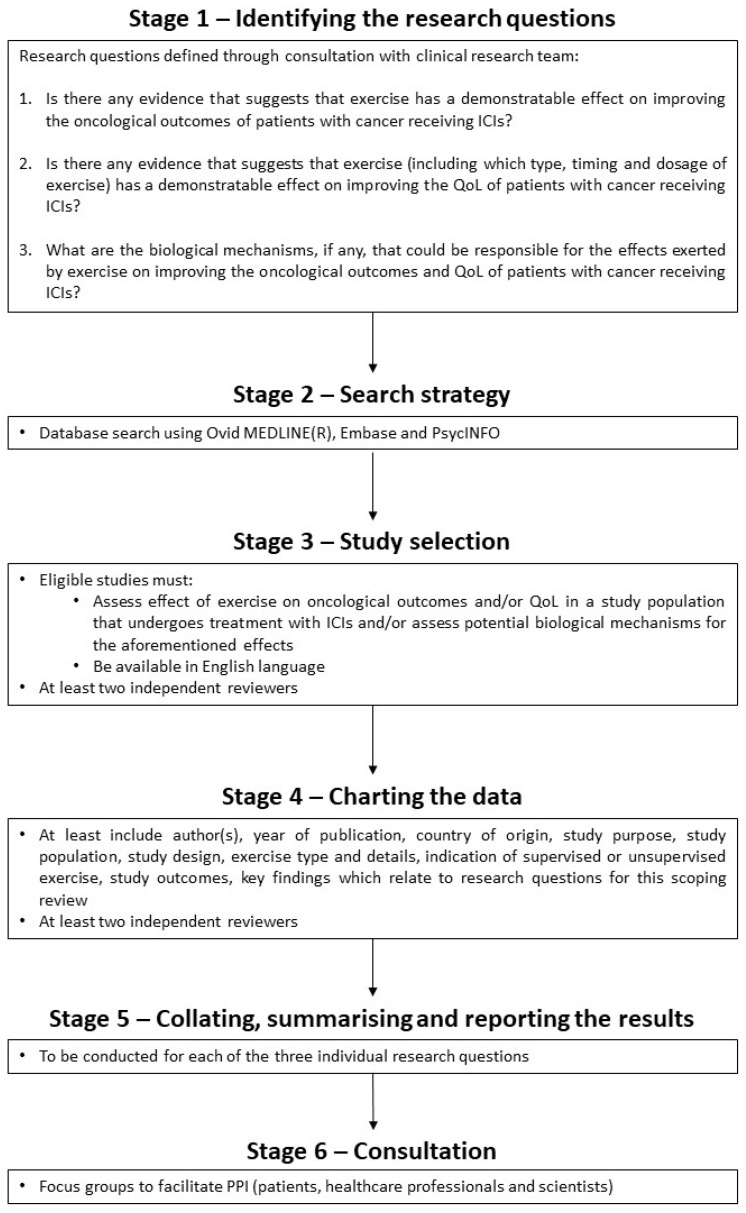
Flowchart of the protocol for this scoping review. Adapted using information from the published protocol [27]. [ICIs, immune checkpoint inhibitors; PPI, patient and public involvement; QoL, quality of life].

We followed the protocol for this scoping review which has been published before [27]. Its development was guided by the Joanna Briggs Institute Manual for Evidence Synthesis [28] and the methodology framework for scoping reviews proposed by Levac et al. [29]. The protocol includes a six-stage methodology (Figure 1). This scoping review has been written up in accordance with the Preferred Reporting Items for Systematic Reviews and Meta-Analyses extension for Scoping Reviews (PRISMA-ScR) checklist [26], which can be found in Table S1 (Supplementary Materials).

### 2.2. Database Search

The OvidSP platform (https://ovidsp.ovid.com/, accessed on 5 April 2022) was used to search for records in EMBASE, APA PsycInfo and Ovid MEDLINE(R), between the inception date of each database and the final search date (5 April 2022). The search strategy can be found in Table S2 (Supplementary Materials). Duplicate records and studies not available in the English language were excluded using the automated tools on OvidSP. 

### 2.3. Identifying Eligible Studies

Remaining records were screened by title and abstract before being reviewed for eligibility. This was conducted by two independent reviewers (JH and BR). Studies were considered eligible if they assessed the effect of exercise on oncological outcomes or QoL in a population who were treated with ICIs for cancer and/or the biological mechanism underpinning such an effect. Studies using outcome measures that were relevant to one or more domains of QoL (e.g., physical functioning and psychological functioning [30]) were considered appropriate for inclusion within the QoL objective of this review. Preclinical studies, observational studies, randomised controlled trials (RCTs) and abstracts were included in the study to provide a broad picture of the current research landscape. 

### 2.4. Charting and Summarising the Data

Data was charted by two independent reviewers according to the variables in Table 1 and recorded in a table using Microsoft Excel. Studies were grouped according to the research question(s) of this scoping review that they addressed. Subsequently, narrative synthesis was used to summarise and report relevant findings for each research question in turn. 

### 2.5. Consultation Phase

The results of the scoping review were presented to, and discussed amongst, a focus group comprising relevant stakeholders such as patient representatives, academics, and healthcare professionals.

## 3. Results

**Figure 2:**
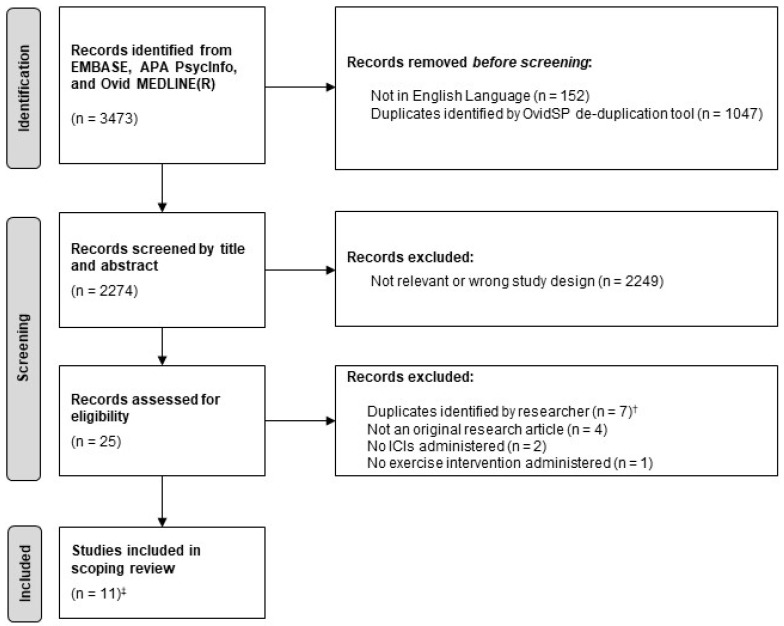
Flowchart of study selection. Adapted using the ‘PRISMA 2020 flow diagram for new systematic reviews which included searches of databases’ template [31]. ^†^ Different records relating to the same study (confirmed by comparison of authors, methodology and reported results) e.g., abstracts from different conferences relating to the same study. Where available, the duplicate record containing a full-text article was included. ^‡^ Two studies were abstract-only. [ICIs, immune checkpoint inhibitors].

Overall, 3473 records were identified in the database search. Duplicate records and those not written in the English language were excluded using an automated tool, leaving 2274 records for screening. Title and abstract screening identified 25 records for eligibility assessment. Of these, 11 studies met the eligibility criteria and were included in this scoping review. The selection process is shown in Figure 2. 

### 3.1. Study Characteristics

The characteristics of the eligible studies (n = 11), of which nine were full-text and two were abstract-only, are shown in Table 2. The studies were conducted across six countries and published between 2017–2021. The majority were preclinical (n = 8), of which seven utilised mouse models and one used a virtual computer model of early-stage cancer. Of the studies in humans (n = 3), one was observational and two were quasi-experimental, utilising a pre-post intervention study design. No RCTs were identified in the search. 

By using a computer model of a virtual cohort of patients, Serrano & Hagar [32] approached exercise and ICI treatment differently to the remaining ten studies, coding aerobic fitness and immune checkpoint blockade within parameters of their model rather than applying direct interventions.

Within the ten remaining studies [33,34,35,36,37,38,39,40,41,42], the type, intensity, duration and level of supervision of the exercise intervention varied, with two studies also administering it alongside additional non-pharmacological interventions [36,39]. Aerobic-only exercise interventions were delivered in the mouse studies, of which three used a supervised approach [33,34,35] and four used an unsupervised approach [36,37,40,41]. Exercise interventions were less prescribed in the clinical studies, each reporting a mixture of aerobic and/or resistance exercise which was supervised and/or unsupervised [38,39,42]. All ten studies used monoclonal antibodies to target the PD-1/PD-L1 axis in at least one subpopulation of their cohort, with three also administering this concurrently with radiotherapy [34], αCTLA-4 [35], or whole tumour-cell vaccines [36]. Melanoma, breast cancer and lung cancer were the most common cancer types specified within the inclusion criteria of the ten studies. Two studies, using preclinical mouse models, delivered the exercise and ICI interventions sequentially [37,40]. The exercise intervention was conducted in the pre-implant setting (i.e., before tumour inoculation), whilst the ICI intervention was delivered in the post-implant setting (i.e., after tumour inoculation). The remaining eight studies included the concurrent delivery of exercise and ICIs, although it should be noted that Turbitt et al. [36] initiated the exercise intervention in the pre-implant setting.

### 3.2. The Effect of Exercise on Oncological Outcomes

Table 3 includes findings from studies addressing the impact of exercise on oncological responses to ICIs (n = 7), all of which were preclinical studies in mouse models of cancer which administered an aerobic-only exercise intervention [33,34,35,36,37,40,41]. The most common outcome measures related to tumour growth.

Four studies provided a direct comparison of oncological outcomes in mice subjected to the combined intervention compared to those subjected to the ICI intervention alone. Of these, only two studies identified a significant improvement in tumour control when combining ICI treatment with exercise [34,35]. Both delivered supervised exercise alongside ICIs in breast cancer models. Wennerberg et al. found that exercise (of unreported intensity) significantly potentiated the response in tumours which were already sensitive to αPD-1 treatment plus radiotherapy [34], whilst Gomes-Santos et al. found that moderate-intensity exercise significantly sensitised tumours which were otherwise unresponsive to ICIs alone (αPD-1 ± αCTLA-4) [35]. By contrast, the other two studies failed to identify a significant effect of exercise on oncological responses to ICIs [33,40]. Similar to Wennerberg et al. and Gomes-Santos et al., Martín-Ruiz et al. also delivered supervised, exercise alongside ICI treatment (αPD-1), albeit in NSCLC mouse models and over a longer period of time [33]. However, moderate-intensity exercise was unable to sensitise tumours to αPD-1, and the combined intervention appeared to provide poorer tumour control compared to αPD-1 alone (although this was not statistically significant) [33]. Post-hoc analyses by Bay et al., who delivered unsupervised exercise before ICI treatment in melanoma models, also showed that exercise was unable to sensitise tumours to αPD-1 [40].

Although their study design permitted, the remaining studies (n = 3) did not provide a direct comparison of oncological responses to the combined approach versus the ICI intervention alone [36,37,41]. Instead, the exercise intervention alone [36,37] and a no intervention control [41] were used as the predominant comparator. No significant differences in oncological outcomes were identified in these studies.

### 3.3. The Effect of Exercise on QoL

Findings from studies addressing the impact of exercise on QoL in the context of ICI treatment (n = 5) are shown in Table 4. Four studies identified at least one benefit of exercise during ICI treatment [35,38,39,42], whilst one study failed to identify any benefit [32].

The clinical studies (n = 3) utilised patient reporting of symptoms and wellbeing as a key outcome measure, either in free-text format [38], or through validated patient-reported outcome measure (PROM) questionnaires [39,42]. Hyatt et al. [38] and Charles et al. [42] both reported improvements in fatigue and mental health after exercise, although the population in Hyatt et al. [38] was not fully described by treatment type and therefore it is unclear whether this cohort had been undergoing ICI treatment as compared to other immunotherapies. By contrast, Lacey et al. [39], who also included dietary and psychological programs alongside exercise as part of a wider supportive care intervention, identified an improvement in memory after the supportive care intervention, but did not identify a clinically meaningful benefit on fatigue or anxiety/depression (a clinically meaningful change for any symptom assessed using the Edmonton Symptom Assessment Scale (ESAS) was defined as a difference of 1 point). In fact, patients reported a worsening in dry mouth. Only Lacey et al. [39] included a measure of overall HRQoL, however this was not shown to have changed by a clinically meaningful amount after the supportive care intervention in patients undergoing ICI treatment.

The preclinical studies (n = 2) used surrogate measures of symptom burden, specifically cancer-related fatigue [35] and treatment related adverse effects [32]. Gomes-Santos et al. [35] showed that, in mice with established cancer undergoing treatment with ICIs, exercise prevented the significant increase in fatigue otherwise seen when mice were subjected to ICI treatment alone. Serrano & Hagar [32] reported that in virtual patients with early-stage cancer, greater aerobic fitness increased the likelihood of treatment-related adverse effects when undergoing ICI treatment and therefore this was the only study which failed to report a benefit of exercise on QoL-related measures. However, it should be noted that the study design, which utilised an in-silico computer model, was somewhat different to the four aforementioned preclinical laboratory-based or clinical studies.

### 3.4. Biological Mechanisms

Table 5 includes the outcome measures and relevant findings from the studies addressing potential biological mechanisms (n = 7). All were preclinical studies in mouse models and were the same seven studies that addressed the impact of aerobic exercise on oncological outcomes when combined with ICI treatment [33,34,35,36,37,40,41].

It was previously noted that Wennerberg et al. [34] and Gomes-Santos et al. [35] observed significantly greater breast tumour control when combining ICI and exercise interventions as compared to the ICI intervention alone (Table 3). As shown in Table 5, both studies found significant exercise-dependent changes in the TIME composition in mice receiving the ICI intervention, albeit in different cell populations. Gomes-Santos et al. [35] observed significantly greater relative and absolute numbers of CD8+ and activated CD8+ T cells in the TIME, whilst Wennerberg et al. [34] identified a significant reduction in the relative numbers of myeloid derived suppressor cells (MDSCs). Wennerberg et al. [34] also highlighted additional potential biological mechanisms, relating to splenic immune cell composition, having observed a significant exercise-dependent increase in the relative number of splenic natural killer (NK) cells and a concurrent reduction in the proportion of splenic NK and CD8+ T cells expressing PD-1 in mice receiving the ICI intervention.

As previously mentioned, Martín-Ruiz et al. [33] and Bay et al. [40] did not observe a significant exercise-dependent change in tumour control when combined with their respective ICI interventions (Table 3). Similarly, neither study reported a significant exercise-dependent change in their measured immune profile. Martín-Ruiz et al. [33] found neither a significant difference in the TIME composition nor a change in the expression of immune checkpoint molecules, whilst Bay et al. [40] found no significant differences in spleen weights or in the cytotoxic capacity of peripheral blood mononuclear cells.

Buss et al. [41] found that exercise-dependent changes in the TIME varied according to tumour type (Table 5). For example, in breast cancer mouse models undergoing αPD-1 treatment, a significant reduction in absolute numbers of CD8+ cells was reported with the incorporation of exercise. By contrast, in mouse melanoma models, a significant increase in the relative number of CD8+ cells was observed. As noted previously, this study did not provide a corresponding comparison of tumour control, therefore the impact of these biological mechanisms on oncological outcomes is unclear.

In line with their approach for the first objective, namely to evaluate any demonstratable effects of exercise on improving the oncological outcomes of patients with cancer receiving ICIs, Turbitt et al. [36] and Unterrainer et al. [37] compared the combined intervention to the exercise intervention alone (rather than ICI treatment alone). It was noted that neither intervention group showed any significant differences in tumour control (Table 3), and indeed Turbitt et al. found no change in splenic MDSC infiltration. Although Unterrainer et al. reported enhanced intratumoural expression of immune checkpoint molecules compared to exercise alone, this cannot be inferred as a significant finding as no *p*-value was quoted.

### 3.5. Consultation Phase

Epidemiologists (n = 2), wet -lab immunologists (n = 2), research assistants (n = 2), an oncologist (n = 1), a patient and public involvement (PPI) coordinator (n = 1), and a patient with cancer who underwent ICI treatment (n = 1), attended the focus group meeting. Discussion predominantly focused on considerations for future clinical research.

The importance of patients feeling motivated and comfortable with the choice of exercise (including type and intensity) was raised as an important factor for ensuring the feasibility of an exercise intervention amongst patients with cancer receiving ICI treatment. It was suggested that it may be helpful for patients to feel in control of the exercise intervention, for it to be independent of a hospital environment, and to receive supervision/support throughout. However, concerns around the feasibility of supervising patients through their exercise intervention, due to the level of resource required, were raised. The focus group highlighted that preference for exercise type may vary by demographic characteristics such as age and gender. Moreover, a potential challenge may be the need to instill significant change in patients’ behaviour, particularly for those who had lived a more sedentary lifestyle. It was suggested that patients may need to feel the benefits of the exercise intervention early on to remain motivated to continue.

Subsequently, the focus group highlighted the need to consider study duration, due to its multifaceted impact on patients’ QoL, patients’ adherence to the exercise intervention, and the ability to measure oncological outcomes and biological changes which manifest over different lengths of time (e.g., short-term inflammatory responses versus long-term immune activation).

Finally, the importance of observing the results of studies assessing the impact of exercise during treatment for cancer, beyond the ICI setting, was raised. The group agreed that the methodologies and results from these studies will be useful for guiding the optimisation of future study design, including the type, intensity, timing, and duration of a exercise intervention in the context of ICI treatment.

## 4. Discussion

Overall, this scoping review identified limited literature addressing each of the three scoping objectives. Most studies were conducted in the preclinical context, with clinical research only assessing the association between exercise and QoL-related factors. Within each objective, studies showed minimal cross-study consensus in methodology and findings. Preclinical mouse studies reported conflicting results as to whether they observed a significant difference in tumour control when mice were subjected to a combined aerobic exercise and ICI intervention versus ICI interventions alone. However, in studies where a significant benefit was observed, a corresponding exercise-dependent shift in the immune profile was reported. Most studies assessing QoL-related factors in the context of ICI treatment concluded at least one benefit of exercise on symptom burden, however the exact effect varied between studies. The consultation phase with multidisciplinary stakeholders identified barriers, facilitators, and logistical factors to consider when designing future clinical projects. The results of this review highlight important gaps in current understanding and areas for future research, both of which will be discussed below.

A previous scoping review, published in 2021, identified four studies addressing the effect of exercise in the context of ICI treatment [43]. Eligible studies were required to deliver concurrent exercise and ICI interventions and to be full-text articles. In line with the current review, the published studies found some evidence to suggest that exercise can reduce treatment-related symptom burden [43]. The current scoping review builds upon the previous work, identifying seven additional studies, by employing wider inclusion criteria to provide a more broad and updated description of the available literature relating exercise to ICIs.

### 4.1. Oncological Outcomes

The first route by which exercise could enhance oncological outcomes is by potentiating the response in ICI-sensitive tumour types. There was a particular paucity of studies addressing this hypothesis. Only Wennerberg et al. [34] did so, showing that aerobic exercise significantly potentiated the response to ICIs (αPD-1) alone in mouse models of triple-negative breast cancer (TNBC). However, whilst the ICI sensitivity observed in this study may, in part, be due to the use of an immunogenic tumour subtype [44], it may also have been enhanced by the use of concurrent radiotherapy. Irradiation has been shown to enhance tumour immunogenicity by promoting the presentation and release of tumour neoantigens, as well as by priming the tumour microenvironment to support infiltration of cytotoxic CD8+ T cells [45,46].

The second route by which exercise could enhance oncological outcomes is by sensitising ICI-resistant tumour types. Excluding triple-negative subtypes, breast cancer is widely characterized as poorly immunogenic and, as such, an unlikely candidate for ICIs [44]. However, Gomes-Santos et al. [35] found aerobic exercise sensitized breast tumours (developed from MCa-M3C cell lines which other studies have characterized to be akin to HER2+ breast cancers [47]) in mice to ICIs. Moreover, in a study published after the search period for this review, Kurz et al. [48] found endurance aerobic exercise sensitized pancreatic ductal adenocarcinomas (a non-immunogenic tumour type which has been characterized as a poor responder to ICI monotherapy [49]) to αPD-1 in mice. Taken together, this suggests there may be a role for aerobic exercise in sensitizing otherwise unresponsive tumours to ICI treatment, but this requires substantiation in wider studies, particularly in the clinical setting. By contrast to the aforementioned studies, Martin-Ruiz et al. [33] and Bay et al. [40] showed that aerobic exercise was unable to sensitise otherwise unresponsive melanoma and NSCLC tumours to ICIs. Differences in immune responses to exercise with, versus without, the concurrent burden of cancer have been observed previously [50]. Therefore the use of pre-implant exercise in Bay et al. [40] may contribute to the difference in findings compared to the aforementioned post-implant studies. The study by Martin-Ruiz et al. [33] is limited by the NSCLC mouse model being devoid of lymphoid cells (the predominant target of ICIs such αPD-1 and a cell population which has been shown to undergo exercise-dependent changes in trafficking and activation in response to aerobic exercise) [41].

Three studies in this review identified no significant benefit of a dual approach versus aerobic exercise (± additional non-pharmacological interventions) interventions alone [36,37,41]. Whilst this may also be indicative of a lack of a significant effect compared to ICI interventions alone, this cannot be directly inferred and, thus, the results are of limited utility when informing upon on the effect of exercise in the context of ICIs. Future studies should be designed to also allow statistical comparisons of oncological outcomes between participants subjected to combined exercise/ICI interventions compared to the ICI intervention alone.

Finally, no studies addressed the impact of resistance or mixed (e.g., aerobic and resistance) exercise interventions on oncological outcomes to ICIs. Resistance exercise is known to influence immune function e.g., through modulation of hormone and cytokine responses (including promoting the release of IL-6 and IL-15 myokines which are associated with trafficking of T-cells and NK-cells to the TIME) [51,52,53,54,55]. Therefore, the role of resistance training as an adjunctive therapy for ICIs holds promise, but this requires elucidation in future research.

### 4.2. Biological Mechanisms

This review found an association between the presence of an aerobic exercise-dependent change in tumour response to ICIs and a corresponding aerobic exercise-dependent change in the immune landscape (of the TIME and/or spleen). In particular, where a significant improvement in tumour control was observed with the combined approach, an exercise-dependent shift towards a more immunogenic landscape occurred. For example, immunosuppressive cell populations which negate cancer responses to immunotherapy were excluded (e.g., MDSCs), whilst potentially pro-inflammatory and cytotoxic cell populations which may help mediate the action of ICIs (e.g., CD8+ T-cells and NK cells) appeared to undergo expansion and activation. These changes are in line with wider literature reporting that bouts of aerobic exercise result in expansion, trafficking and activation of tumour-suppressive immune cell populations, in particular lymphocyte subsets such as NK cells, CD8+ T-cells and γδ T-cells [24,48,50,51].

Few studies in this review considered biological outcome measures beyond the quantification of relative trafficking and activation of leukocyte populations. However, previous literature has outlined a diverse range of mechanisms by which exercise can influence immune outcomes [24]. Aerobic exercise causes changes in hormone profiles, such as increased release of cortisol and catecholamines [24,56]. With respect to the latter, exercise-induced epinephrine release has been found to promote NK-cell and CD8+ cell mobilisation through β-adrenergic signalling pathways [24,51]. Acute aerobic exercise and resistance exercise also promote the release of myokines (muscle-derived cytokines) into circulation, including IL-15 and IL-6, which are thought to aid the trafficking of lymphocyte populations to the TIME [24,48,51]. Indeed, blocking β-adrenergic pathways or IL-6 function in tumour-bearing mice dampened the aerobic exercise-dependent suppression of tumour growth [18]. Exercise also influences factors which affect the immune system via more indirect processes. This includes systemic changes (such as altered levels of adiposity) and changes which are more localised to tumours (such as reduced hypoxia and improved blood vessel normalisation) [18,24,56]. It should be noted that different exercise regimens may result in differing immune responses. For example, greater intensity and duration of exercise was shown to cause an exponential increase in levels of IL-6 in peripheral blood [24]. Moreover, expanded populations of NK cells return to baseline levels within 24 h of an acute bout of aerobic exercise [50], whereas chronic exercise (repetitive bouts of exercise over a short-term or long-term period) is thought to result in more sustained adaptations within the haematopoietic system [25]. The roles of these biological mechanisms, and the relative effects of different exercise regimens, should be addressed in future studies considering the role of exercise in the context of ICIs.

### 4.3. QoL

Overall, the studies in this review reported heterogeneous effects of exercise on a range of different symptom measures. This may, in part, be due to the varied exercise interventions undertaken by study participants. However, most studies reporting upon fatigue (or a preclinical surrogate of this outcome) found that exercise reduced fatigue during ICI treatment. This is in line with a wider body of literature which found exercise to be effective at reducing cancer- and treatment-related fatigue [10,11,12]. In a meta-analysis of over 100 studies, exercise was significantly more effective than pharmacological agents targeting fatigue [57]. Given that fatigue is the most commonly reported adverse events across studies incorporating αPD-1 or anti-PDL1 (αPD-L1) interventions [58], these findings emphasise the potential benefit of exercise for symptom burden if delivered alongside ICIs and this warrants larger, more comprehensive, investigations.

Most studies addressing QoL used outcome measures relating to symptom burden, with only Lacey et al. [39] incorporating a global measure of HRQoL. Although the authors identified clinically meaningful improvements in particular symptoms with the uptake of exercise, there was no significant change in general HRQoL [39]. Whist this trend is difficult to robustly conclude from a single study, the findings are corroborated by a wider body of evidence. For example, although symptom burden has been shown to associate with QoL measures in patients with cancer generally [59,60], the association can be poor in those receiving ICIs [61]. This may, in part, be caused by HRQoL PROMs failing to capture the full range of symptoms experienced by patients undergoing treatment with ICIs. There have been calls for novel PROMs to be developed for use in the context of ICIs [61]; an important consideration for the design of future clinical research assessing the impact of exercise on QoL during ICI treatment.

### 4.4. Clinical Research

We did not find any clinical studies addressing the objectives relating to oncological outcomes or biological mechanisms. Whilst preclinical studies are useful, mouse models of cancer are unlikely to fully recapitulate the complexity and variability of malignancies in the clinical context. Moreover, the preclinical studies were unable to assess long-term oncological outcomes as they were required to terminate experiments before tumours extended beyond a maximum ethical size. Sportivumab [62] and HI AIM [63] are ongoing trials delivering exercise programmes for patients with melanoma and NSCLC, respectively, being treated with ICIs. With endpoints including survival outcomes, QoL and changes in the immune landscape, the results of these trials will help inform upon the impact of exercise during ICI treatment in the clinical setting.

### 4.5. Strengths and Limitations

By employing a broad inclusion criterion (which included preclinical studies, abstract-only studies, and by not specifying the need for concurrent interventions), a comprehensive summary of the available literature relating exercise and ICIs could be provided, reducing reporting bias. Although the consultation phase considered a range of perspectives which facilitated a broad discussion about future research, additional perspectives may have been helpful to include; specifically, exercise scientists and oncologists representing the tumour types most frequently included by the studies within this review. Abstract-only studies may be subject to ambiguity in the interpretation of findings, due to the limited information presented on methodology and results. Quantitative synthesis was not feasible due to the limited number of studies and the significant heterogeneity in malignant disease settings and outcome measures. Moreover, differences in the methodology of eligible studies prevented robust conclusions regarding the impact of exercise on cancer outcomes in the context of ICIs. As a result, recommendations to inform the inclusion of exercise alongside ICIs in practice (in particular the optimal type, timing and intensity to improve patient outcomes) could not be drawn.

## 5. Conclusions

Overall, this scoping review shows that the effect of exercise in the context of ICIs remains an under-researched field. Although this review hypothesised that exercise can improve oncological outcomes and QoL during ICI treatment, a clear conclusion could not be reached from the current literature in the field, due to the considerable heterogeneity in the methodology and findings. Future work is required, particularly within the clinical setting, to increase the evidence base and facilitate the development of robust guidelines for prescribing exercise for patients receiving ICIs across solid and haematological tumour types.

## Figures and Tables

**Table 1:** Variables for which data were sought from eligible studies.

Variable	Definition
Authors	Name(s) of the author(s) of the study
Country	Country in which study was conducted
Year	Year of publication
Study design	Broad study type (e.g., preclinical/observational/quasi-experimental/randomised controlled trial)
Population	Total number and characteristics of study participants
Purpose	Aim(s) of the study
Cancer	Tumour type(s) represented amongst the study population
ICI	Method of immune checkpoint blockade, including any other oncological intervention delivered concurrently
Exercise intervention	Details of the exercise intervention, including type (e.g., aerobic/resistance/mixed), intensity, frequency and any other supportive care intervention delivered concurrently
Exercise intervention oversight	Level of supervision of the exercise intervention (e.g., supervised/unsupervised/mixed)
Concurrent exercise and ICI	Indication of whether the study included any concurrent delivery of the exercise and ICI interventions or not (e.g., yes/no)
Scoping objective(s)	The research question(s) of the present scoping review addressed by the study
Outcome measure(s)	The endpoints used in the study which relate to the research question(s) of the present scoping review
Relevant findings	Results of the study which relate to the research questions(s) of the present scoping review

ICI, immune checkpoint inhibitor.

**Table 2:** Characteristics of eligible studies (n = 11).

Authors, Country, Year	Study Design, Population	Purpose	Cancer	ICI	Exercise Intervention	Exercise Intervention Oversight	Concurrent Exercise and ICI	Scoping Objectives ^1^	Ref.
Serrano & Hagar, USA, 2021	Preclinical (in silico computer model) Virtual cohort of patients with early-stage solid tumour cancer (n = 200)	To describe an in-silico model simulating early-stage solid tumour growth and anti-tumour immune response, and demonstrate its utility through two virtual experiments.	Solid tumour cancer	Treatment with ICIs was modelled by reducing the inhibitory parameter of Tregs, resulting in increased efficacy of cytotoxic T lymphocytes.	Aerobic fitness was a pre-existing parameter in the virtual model. The model used clinical and epidemiological data on aerobic fitness to divide the virtual cohort into aerobically fit or sedentary groups.	N/A	N/A	2	[32]
Martin-Ruiz et al., Spain, 2020	Preclinical Mouse tumour model (n = 22)	To determine the effects of exercise on tumour growth and its potential adjuvant effects when combined with αPD-1 immunotherapy (nivolumab).	NSCLC	αPD-1	Mixed (combination of aerobic and resistance). Moderate intensity. 40–60 min per session. 5 days per week for 8 weeks.	Supervised	Yes (Post-implant exercise initiated when tumour reached 100 mm^3^. ICI initiated 15 days later. ICI and exercise were then delivered concurrently for 6 weeks)	1, 3	[33]
Wennerberg et al., USA, 2020	Preclinical Mouse tumour model (n = 42)	To determine whether exercise has anti-tumour effects in a model of established triple-negative breast cancer.	Breast cancer (triple negative)	αPD-1 (+RT)	Aerobic (treadmill running). 30 min per session. 5 days/week for ~3 weeks.	Supervised	Yes (Post-implant exercise initiated 8 days after tumour inoculation and continued until end of experiment. αPD-1 delivered on day 15, 19 and 23)	1, 3	[34]
Gomes-Santos et al., USA, 2021	Preclinical Mouse tumour model (total n not explicity specified)	To determine whether CD8+ T-cells mediate effect of exercise on tumour control, and to examine effect of exercise on response to immunotherapy.	Breast cancer	αPD-1 ± αCTLA-4	Aerobic (treadmill running). Moderate-vigorous intensity. 30–45 min per session. 5 days per week for 1–2 weeks.	Supervised	Yes (Exercise and ICI interventions initiated concomitantly when tumours reached prespecified volume)	1, 2, 3	[35]
Turbitt et al., USA, 2017	Preclinical Mouse tumour model (n = 64)	To determine whether preventing weight gain (through diet and exercise interventions) improves responses to whole tumour cell vaccine and PD-1 checkpoint blockade.	Breast cancer	αPD-1 (±whole tumour cell vaccine)	Aerobic (voluntary wheel running). Range of distance run not reported. Continued for 8 weeks + 35 days. Delivered alongside a dietary intervention (10% reduction in calorie intake).	Unsupervised	Yes (8 weeks of pre-implant exercise plus 35 days of post-implant exercise. Whole tumour cell vaccine initiated 7 days post-implant and αPD-1 initiated 9–12 days post-implant)	1, 3	[36] ^2^
Unterrainer et al., Denmark, 2018	Preclinical Mouse tumour model (n not provided)	To determine whether exercise enhances responses to ICIs given an exercise-dependent increase in intratumoural immune cell infiltration.	Melanoma	αPD-L1	Aerobic (voluntary wheel running). Ranged from 1.8–5.4 km per day for 4 weeks.	Unsupervised	No (4 weeks of pre-implant exercise followed by αPD-L1, which was initiated 4 days post-implant)	1, 3	[37] ^2^
Hyatt et al., Australia, 2019	Observational (survey) Patients with unresectable stage 3/4 melanoma receiving immunotherapy (n = 55)	To describe the levels of fatigue, exercise behaviours, and barriers/facilitators to engaging with exercise in patients receiving immunotherapy for melanoma.	Melanoma	Mixed (n = 8 known to have received αPD-1 or αCTLA-4, however no data provided on remainder of cohort)	Mixed (exercise regimes were individual to each patient and were aerobic or a combination of aerobic/resistance). Of those who exercised during immunotherapy treatment (n = 31), most spent <60 min per session and exercised <5 times per week.	Mixed	Yes (Participants were asked to recall the impact of exercise, if undertaken, whilst they were undergoing immunotherapy)	2	[38]
Lacey et al., Australia, 2019	Quasi-experimental (pre-post test) Patients with metastatic melanoma receiving pembrolizumab (n = 28)	To assess the feasibility of a multimodal supportive care intervention in patients with metastatic melanoma receiving pembrolizumab.	Melanoma	αPD-1	Mixed (exercise regimes were individual to each patient and were aerobic, resistance, or a combination of modes). 16 exercise sessions over 8 weeks. Delivered as part of a wider holistic supportive care program (including dietary advice, non-invasive complementary therapies and psychological consultation).	Mixed	Yes (Exercise intervention prospectively delivered in participants who were also undergoing ICI treatment)	2	[39]
Bay et al., Denmark, 2020	Preclinical Mouse tumour model (n = 112)	To determine whether voluntary wheel running leads to increased expression of checkpoint molecules and achieves an additive effect when combined with ICIs.	Mixed (Melanoma, breast cancer, LLC, HNSCC)	αPD-1 or αPD-L1	Aerobic (voluntary wheel running). Ranged from 4–8 km per day (in αPD-1 experiment) or 0.9–5.8 km per day (in αPD-L1 experiment) for 5 weeks.	Unsupervised	No (5 weeks of pre-implant exercise followed by αPD-1 or αPD-L1, which was initiated 4 days post-implant)	1, 3	[40]
Buss et al., New Zealand, 2021	Preclinical Mouse tumour model (n = 104)	To determine whether exercise after introduction of cancer cells enhances efficacy of concurrent αPD-1 treatment and increases infiltration of cytotoxic immune cells, improves perfusion, and reduces hypoxia.	Mixed (Melanoma, breast cancer)	αPD-1	Aerobic (voluntary wheel running). Range of distance run not reported. Continued until tumours reached maximum ethical size (2–5 weeks, median 19 days)	Unsupervised	Yes (Exercise and αPD-1 interventions initiated post-implant and continued until tumours reached maximum ethical size)	1, 3	[41]
Charles et al., France, 2021	Quasi-experimental (pre-post test) Patients with cancer undergoing treatment with ICIs, and presenting with moderate to severe fatigue (n = 16)	Firstly, to determine whether a 6-month videoconference programme promoting exercise is feasible in patients with cancer undergoing immunotherapy and, secondly, to assess whether exercise reduces patients’ fatigue.	Mixed (melanoma, lung cancer, other)	αPD-1	Mixed (combination of aerobic, resistance, other). Aimed for at least 150 min of exercise per week for 6 months.	Mixed	Yes (Exercise intervention prospectively delivered in participants who were also undergoing ICI treatment)	2	[42]

αCTLA-4, anti cytotoxic T-lymphocyte associated antigen 4; αPD-1, anti programmed cell death protein 1; αPD-L1, anti programmed cell death-ligand 1; HNSCC, head and neck squamous cell carcinoma; ICI(s), immune checkpoint inhibitor(s); LLC, Lewis Lung Cancer; N/A, not applicable; NSCLC, non-small cell lung cancer; RT, radiotherapy; Tregs, regulatory T-cells; USA, United States of America. ^1^ 1, “Is there any evidence that suggests that exercise has a demonstratable effect on improving the oncological outcomes of patients with cancer receiving ICIs?”; 2, “Is there any evidence that suggests that exercise (including which type, timing and dosage of exercise) has a demonstratable effect on improving the QoL of patients with cancer receiving ICIs?”; 3, “What are the biological mechanisms, if any, that could be responsible for the effects exerted by exercise on improving the oncological outcomes and QoL of patients with cancer receiving ICIs?”; ^2^ Abstract only.

**Table 3:** Findings from studies addressing the effect of exercise on oncological responses to ICIs (n = 7).

Authors	Outcome Measure(s)	Relevant Findings	Ref.
Martin-Ruiz et al.	Final tumour volume, percentage change in tumour volume, necrotic index, cell proliferation index, apoptotic index	Exercise, but not ICI treatment (αPD-1), alone significantly suppressed tumour growth. Combining ICI treatment with exercise did not result in a significant difference in tumour growth, necrosis, apoptosis, or cell proliferation compared to ICI intervention alone.	[33]
Wennerberg et al.	Tumour volume over time, final tumour volume, metastatic burden in lungs	Exercise alone and ICI treatment (αPD-1+RT) alone significantly suppressed tumour growth. Moreover, combining ICI treatment with exercise further enhanced the response, with significantly slowed tumour growth compared to ICI treatment alone.	[34]
Gomes-Santos et al.	Tumour volume over time, final tumour volume	Exercise alone significantly suppressed tumour growth (according to final tumour volume), however ICI treatment (αPD-1 ± αCTLA-4) did not. Combining ICI treatment with exercise sensitised established breast cancer tumours to ICIs, with significantly greater tumour suppression compared to ICI treatment alone.	[35]
Turbitt et al.	Final tumour volume, final tumour weight, metastatic burden in lungs	The weight management intervention (exercise + calorie restriction) alone suppressed tumour growth. Compared to the weight management intervention alone, no statistically significant benefit on tumour control or metastatic burden was observed when combined with αPD-1 or αPD-1 + whole tumour cell vaccine, respectively.	[36] ^1^
Unterrainer et al.	Tumour growth (not otherwise specified)	Exercise alone had an overall suppressive effect. Combining ICI treatment (αPD-L1) and exercise appeared to suppress tumour growth more than exercise alone, but this was not a statistically significant effect.	[37] ^1^
Bay et al.	Tumour volume over time, final tumour weight	Exercise, but not ICI treatment (αPD-1 or αPD-L1), alone significantly suppressed tumour growth. Combining ICI treatment and exercise did not result in a statistically significant additive effect on tumour control compared to exercise alone. A non-significant trend towards greater tumour control with αPD-1+exercise compared to αPD-1 alone was observed.	[40]
Buss et al.	Tumour growth (not otherwise specified)	Exercise alone, ICI treatment (αPD-1) alone, nor their combination significantly altered tumour growth rate compared to sedentary, isotype control treated mice.	[41]

αCTLA-4, anti cytotoxic T-lymphocyte associated antigen 4; αPD-1, anti programmed cell death protein 1; αPD-L1, anti programmed cell death-ligand 1; ICI(s), immune checkpoint inhibitor(s); RT, radiotherapy. ^1^ Abstract only.

**Table 4:** Findings from studies addressing the impact of exercise on QoL in the context of ICIs (n = 5).

Authors	Outcome Measure(s)	Relevant Findings	Ref.
Serrano & Hagar ^1^	Cytotoxicity parameter in model (surrogate for treatment related adverse effects)	Virtual patients who were aerobically fit were more likely to experience adverse effects when receiving the same dose of ICI as compared to sedentary patients.	[32]
Gomes-Santos et al. ^1^	Exercise capacity (surrogate for fatigue)	Mice with established cancer undergoing ICI treatment alone (αPD-1 ± αCTLA-4) had significantly reduced exercise capacity (i.e., increased fatigue) compared to normal controls. However, combining ICI treatment with an exercise intervention restored exercise capacity (i.e., prevented the increase in fatigue).	[35]
Hyatt et al.	Patient-reported free-text responses to survey questions	In patients who exercised during immunotherapy (n = 31), many reported benefits of exercise with the most common being reduced treatment-related fatigue. Other benefits included increased energy, improved overall wellbeing, improved sleep, and improved mental health.	[38]
Lacey et al.	PROM questionnaires	In patients who carried out exercise (as part of a wider holistic supportive care intervention) alongside ICI treatment (αPD-1) (n = 13), a clinically meaningful improvement in memory but worsening in dry mouth was observed where comparing post- vs. pre- supportive care intervention. There was no clinically meaningful change in anxiety/depression or general HRQoL.	[39]
Charles et al.	PROM questionnaires	For patients receiving ICIs (αPD-1) who completed the pre- and post- exercise intervention assessments (n = 13), a significant improvement in fatigue was observed as well as improvement in overall perception of physical and mental health.	[42]

αCTLA-4, anti cytotoxic T-lymphocyte associated antigen 4; αPD-1, anti programmed cell death protein 1; HRQoL, health-related quality of life; ICI(s), immune checkpoint inhibitor(s); PROM, patient-reported outcome measure. ^1^ Preclinical studies which were deemed appropriate to include within the QoL objective of this review as their outcome measures related to one or more domains of QoL [30].

**Table 5:** Findings from studies addressing biological mechanisms (n = 7).

Authors	Outcome Measure(s)	Relevant Findings	Ref.
Martin-Ruiz et al.	TIME composition (all leukocytes, neutrophils, monocytes/eosinophils), expression of murine genes relating to the immune system	Combining ICI treatment (αPD-1) with exercise did not result in a significant difference in intratumoural leukocyte, neutrophil, or monocyte/eosinophil infiltration when compared to ICI treatment alone. ICI treatment alone, or in combination with exercise, resulted in significantly increased intratumoural vegf-a expression compared to exercise alone.	[33]
Wennerberg et al.	TIME composition (MDSCs), splenic composition (CD8+ T-cells and NK cells), expression of Ki67, CD69, PD-1	Combining ICI treatment (αPD-1+RT) with exercise significantly reduced intratumoural MDSC infiltration, increased splenic NK cell infiltration, and reduced PD-1 expression on splenic NK and T cells compared to ICI treatment alone.	[34]
Gomes-Santos et al.	TIME composition (CD8+ T-cells, activated CD8+ T-cells)	Combining ICI treatment (αPD-1 ± αCTLA-4) with exercise synergistically increased the proportion of activated CD8+ T-cells compared to ICI treatment alone.	[35]
Turbitt et al.	Splenic composition (MDSCs)	Combining ICI treatment (αPD-1 + whole tumour cell vaccine) with a weight management intervention (exercise + calorie restriction) did not significantly affect splenic MDSC infiltration compared to the weight management intervention alone.	[36] ^1^
Unterrainer et al.	TIME composition (cytotoxic NK cells and T-cells), expression of immune checkpoint molecules	Exercise alone increased intratumoural expression of immune checkpoint molecules (PD-L1, B7.1, B7.2, PD-1, CD28) and infiltration of cytotoxic NK and T-cells (*p*-value not provided). Combining ICI treatment (αPD-L1) with exercise enhanced intratumoural expression of immune checkpoint molecules and receptors compared to exercise alone (*p*-value not provided).	[37] ^1^
Bay et al.	Expression of immune checkpoint molecules, spleen weight, killing capacity of PBMCs	Exercise alone significantly increased intratumoural expression of immune checkpoint molecules (PD1, PD-L1, PD-L2, B7.1, B7.2 and CD28) in the melanoma mouse model. Only PD-L1 and CD28 were upregulated in the LLC model, and none were upregulated in the breast cancer model. Combining ICI treatment (αPD-1 or αPD-L1) with exercise showed no significant difference in spleen weight or PMBC killing capacity compared to ICI treatment or exercise alone.	[40]
Buss et al.	TIME infiltration (NK cells and T-cells), hypoxia, tumour perfusion	Combining ICI treatment (αPD-1) with exercise significantly reduced absolute numbers of tumour-infiltrating CD8+ cells (in breast tumours) but significantly increased the relative number of CD8+ cells (in melanomas) compared to treatment with ICIs alone.	[41]

αCTLA-4, anti cytotoxic T-lymphocyte associated antigen 4; αPD-1, anti programmed cell death protein 1; αPD-L1, anti programmed cell death-ligand 1; ICI(s), immune checkpoint inhibitor(s); LLC, Lewis lung cancer; MDSC, myeloid derived suppressor cell; NK, natural killer; PBMC, peripheral blood mononuclear cells; RT, radiotherapy; TIME, tumour immune microenvironment. ^1^ Abstract only.

## Supplementary Materials

The following supporting information can be downloaded at: https://www.mdpi.com/article/10.3390/cancers14205039/s1, Table S1: PRISMA-ScR checklist; Table S2: Search strategy.
