# Supplementary material for: Is There a Role for Exercise When Treating Patients with Cancer with Immune Checkpoint Inhibitors? A Scoping Review
Source: Cancers (Basel). 2022 Oct 14;14(20):5039. doi: 10.3390/cancers14205039 (PMC9599872; doi:10.3390/cancers14205039)
Supplement: Supplementary file 1 [file cancers-14-05039-s001.zip › Table S2_ProofreadComplete.pdf]

cancers

MDPI

Table S2. Search strategy.

**Objective 1 and 2**	
1	exp Neoplasms/ or exp malignant neoplasm/
2	(cancer or tumour or tumor or malignan* or oncolog* or neoplas*).kw,tw.
3	1 or 2
4	exp Exercise/ or exp Aerobic exercise/
5	(exercis* or (physical* adj1 (activ* or fitness or fit or training)) or cycling or running or aerobic or anaerobic or resistance exercis* or swimming).kw,tw.
6	4 or 5
7	Immunotherapy/ or cancer immunotherapy/
8	(immunotherapy or immun* therapy or immune checkpoint inhibitors or ((PD-1 or PD1 or PD-L1 or PDL1 or CTLA-4 or CTLA4) adj3 inhibit*) or monoclonal antibod* or nivolumab or opdivo or pembrolizumab or keytruda or ipilimumab or yervoy or atezolizumab or MPDL3280A).kw,tw.
9	7 or 8
10	3 and 6 and 9
11	remove duplicates from 10
**Objective 3**	
1	exp Neoplasms/ or exp malignant neoplasm/
2	(cancer or tumour or tumor or malignan* or oncolog* or neoplas*).kw,tw.
3	1 or 2
4	exp Exercise/ or exp Aerobic exercise/
5	(exercis* or (physical* adj1 (activ* or fitness or fit or training)) or cycling or running or treadmill or aerobic or anaerobic or resistance exercis* or swimming).kw,tw.
6	4 or 5
7	(immun*).ti.
8	3 and 6 and 7
9	remove duplicates from 8
